# A novel nutritional index for predicting stroke-heart syndrome and clinical outcomes after endovascular treatment

**DOI:** 10.3389/fnut.2026.1822338

**Published:** 2026-05-18

**Authors:** Wenjie Chen, Yahang Tan, Xuesong Bai, Tao Wang, Liqun Jiao, Liyong Zhang, Hong Li

**Affiliations:** 1 Department of Cardiology, Beijing Chaoyang Hospital, Beijing, China; 2 Department of Neurosurgery, Beijing Xuanwu Hospital, Beijing, China; 3 Department of Neurosurgery, Liaocheng People's Hospital, Liaocheng, Shandong Province, China

**Keywords:** acute ischemic stroke, endovascular thrombectomy, myocardial injury, stroke-heart syndrome, triglyceride-cholesterol-body weight index

## Abstract

**Background:**

Metabolic-nutritional reserves influence stroke outcomes, but their role in large vessel occlusion acute ischemic stroke (LVO-AIS) treated with endovascular thrombectomy (EVT) remains unclear. We investigated whether the triglyceride-cholesterol-body weight index (TCBI) predicts dynamic myocardial injury after stroke and 90-day functional outcomes.

**Methods:**

This two-center retrospective cohort study included 524 anterior-circulation LVO-AIS patients undergoing EVT (2021–2024). TCBI was calculated as (triglycerides × total cholesterol × body weight)/1,000. Myocardial injury was classified using serial cardiac troponin I (cTnI) measurements into no injury, non-dynamic elevation, or dynamic elevation. The primary outcome was poor 90-day functional outcome (modified Rankin Scale [mRS] 3–6). Ordered logistic regression, restricted cubic splines, and the mediation analysis were performed.

**Results:**

A total of 524 patients were included. Low TCBI (≤1244) was associated with more severe myocardial injury (OR = 1.67, 95% CI: 1.13–2.47, *p* = 0.010) and 90-day poor functional outcome (OR = 1.54, 95% CI: 1.06–2.23, *p* = 0.024). TCBI predicted poor functional outcome with an AUC of 0.723 (95% CI: 0.680–0.766), significantly outperforming albumin, TG, and BMI (all DeLong, *p* < 0.001). In exploratory mediation analysis, myocardial injury mediated 45.6% of the total effect of low TCBI on poor outcomes (ACME *p* = 0.004). PSM analyses yielded consistent results.

**Conclusion:**

Low TCBI was independently associated with stroke-induced myocardial injury and poor 90-day functional outcomes in LVO-AIS patients undergoing EVT, with myocardial injury serving as a potential mediator. These findings suggest that nutritional status may play a role in post-stroke cardiac and functional recovery, warranting further prospective investigation.

## 1 Introduction

Although endovascular thrombectomy (EVT) has achieved a high rate of vascular recanalization, a considerable proportion of patients with large vessel occlusion acute ischemic stroke (LVO-AIS) still face poor prognosis (1). Stroke-Heart Syndrome (SHS) is one of the main drivers of poor prognosis (2, 3). Among the various manifestations of SHS, myocardial injury is particularly common. Existing evidence indicates that a single measurement of troponin is often insufficient to assess risk; instead, the dynamic trajectory of troponin changes is better at distinguishing acute ischemic injury from chronic stable elevation, thereby providing better predictive value (4). However, it remains unclear which patients are more prone to this dynamic myocardial injury after stroke.

Nutritional status plays a critical role in the body’s response to acute stress. Malnutrition is associated with increased mortality and complications in patients with acute ischemic stroke (5). Established nutritional indices (GNRI, CONUT, PNI) predict outcomes in cardiovascular diseases (6, 7), but rely on serum albumin, which fluctuates during acute inflammation (8). Recently, a novel composite index integrating triglycerides, total cholesterol, and body weight - the triglyceride-cholesterol-body weight index (TCBI) - has been proposed as an objective indicator for assessing systemic lipid-energy reserves and has shown superior predictive value compared to single indicators in heart failure and general stroke populations (9–12). However, the current evidence still has significant limitations. Most existing studies have focused on general stroke populations, lacking data on the high-risk subgroup of LVO patients treated with EVT. The mechanism by which low TCBI leads to poor prognosis remains unclear.

Therefore, this study aims to evaluate the association between TCBI and dynamic myocardial injury in LVO patients treated with EVT based on two-center data, and further explore whether myocardial injury plays a mediating role in the relationship between low TCBI and poor functional prognosis.

## 2 Materials and methods

### 2.1 Study design and population

This retrospective, two-center cohort study examined data from consecutive adult patients who underwent EVT for anterior-circulation LVO-AIS from June 2021 through June 2024. The inclusion criteria were: (1) minimum age of 18 years; (2) pre-stroke modified Rankin Scale (mRS) score of 2 or less; (3) occlusion of the intracranial internal carotid artery (ICA) or middle cerebral artery (M1/M2 segments) confirmed by digital subtraction angiography (DSA) or CT/MR angiography; (4) EVT initiation within 6 h of symptom onset, or within 6–24 h meeting the DAWN/DEFUSE-3 eligibility criteria (1, 13).

The exclusion criteria were: (1) presence of active malignancy or severe infection at admission, to avoid confounding effects on inflammatory biomarkers; (2) concurrent acute coronary syndrome (ACS), to distinguish stroke-associated myocardial injury from primary myocardial infarction; (3) known chronic kidney disease (CKD) or severe renal insufficiency (defined as serum creatinine >2.0 mg/dL, eGFR <30 mL/min/1.73m^2^, or maintenance dialysis), given that impaired renal clearance can lead to non-specific troponin elevation; and (4) missing data for key variables (components of the TCBI index) or failure to complete 90-day follow-up. The study received approval from the institutional review boards of both participating centers, and the requirement for written informed consent was waived due to the retrospective nature of the analysis.

### 2.2 Clinical and procedural data collection

Patient demographic and clinical characteristics at baseline were obtained from prospectively maintained stroke registries and complemented by review of the electronic medical record. We recorded age, sex, and comorbidities (hypertension, coronary artery disease (CAD), diabetes mellitus, atrial fibrillation (AF), hyperlipidemia, prior stroke), and smoking status. Stroke severity at baseline was quantified using the National Institutes of Health Stroke Scale (NIHSS). Baseline infarct core was estimated with the Alberta Stroke Program Early CT Score (ASPECTS) on non-contrast CT (14).

The cause of stroke was adjudicated according to the TOAST classification and grouped as cardioembolism, large-artery atherosclerosis, or other causes (15, 16). The occlusion location was defined on baseline angiographic imaging and categorized as ICA, M1, or M2 segment occlusion (17). Procedural data included the first-line thrombectomy strategy (no device pass, aspiration, stent retriever, or a combined approach) and final reperfusion graded by the modified Thrombolysis in Cerebral Infarction (mTICI) score (18). Successful reperfusion was defined as mTICI 2b-3 (19, 20). Periprocedural complications included symptomatic intracranial hemorrhage (sICH) and malignant cerebral edema (21). sICH was diagnosed according to the Heidelberg Bleeding Classification (22–24). Malignant cerebral edema was defined as: (1) hypodense parenchymal involvement in at least 50% of the MCA territory, regional brain swelling or cerebral herniation and (2) a ≥ 5 mm midline shift at the septum pellucidum or pineal gland, with effacement of the basal cisterns (25).

Lipid profiles, including triglycerides (TG, mg/dL) and total cholesterol (TC, mg/dL), were measured using standard enzymatic methods. TG and TC were measured from venous blood samples collected on the morning following admission, after an overnight fast of at least 8 h. Fasting blood samples were obtained at a median of 17 h (IQR 13–23 h) post-admission. All fasting samples were collected after EVT completion (admission to EVT initiation: 1.5 h (1.2–2.1)). Body weight (BW, kg) was measured at admission using a calibrated electronic scale. The triglyceride-cholesterol-body weight index (TCBI) was calculated as: TCBI = (TG × TC × BW) / 1,000 (5).

### 2.3 Definition of troponin trajectory

cTnI was measured using a high-sensitivity assay at two timepoints: at admission (pre-EVT) and at 72 h after admission. Both participating centers used the same high-sensitivity assay platform. The institutional upper reference limit (URL, 99th percentile) was 0.034 ng/mL. To characterize the phenotype of SHS, troponin trajectories were classified into three ordinal categories based on the dynamic change between the two time points:

No Myocardial Injury: All cTnI measurements remained ≤ URL;

Non-Dynamic Elevation (Chronic/Stable Injury): At least one cTnI measurement > URL, but without a significant rise. This category included patients with stable elevation (change <20%) or a falling pattern (decrease ≥20% from admission to 72 h), reflecting chronic myocardial injury or resolving acute injury preceding the index stroke;

Dynamic Elevation (Acute Injury): At least one cTnI measurement > URL, with a distinct rising pattern (≥20% increase from admission to 72 h). This pattern is pathognomonic of acute, evolving myocardial injury potentially triggered by the stroke event (Stroke-Heart Syndrome).

The 20% relative change threshold was adopted from the Fourth Universal Definition of Myocardial Infarction to distinguish acute from chronic injury (2).

### 2.4 Outcome measures

The primary outcome was poor functional outcome at 90 days, defined as mRS score of 3–6 (moderate-to-severe disability or death). Functional outcomes were assessed by certified stroke nurses or physicians during in-person clinic visits or structured telephone interviews at 90 ± 14 days after stroke onset. Assessors were blinded to patients’ troponin trajectory classification and TCBI values.

### 2.5 Statistical analysis

Patients with missing data for TCBI components were excluded. 90-day functional outcome was available for all included patients, with no loss to follow-up. Baseline characteristics were summarized across the three myocardial injury groups. Normality of continuous variables was assessed using the Shapiro–Wilk test. Normally distributed continuous variables are presented as mean ± standard deviation; non-normally distributed variables are presented as median (interquartile range [IQR]). Differences among groups were compared using the Kruskal–Wallis test for continuous variables and chi-square test or Fisher’s exact test for categorical variables, as appropriate.

The potential nonlinear association between TCBI and myocardial injury severity was evaluated using restricted cubic spline (RCS) analysis within an ordered logistic regression model. Four knots were placed at the 5th, 35th, 65th, and 95th percentiles of the TCBI distribution. The overall association and departure from linearity were assessed using likelihood ratio tests.

To characterize the threshold pattern suggested by the spline curve, a candidate cutoff was selected based on the approximate crossing point of the RCS-estimated odds ratio curve with the null reference line, together with previously reported threshold ranges. A two-piecewise ordered logistic regression model was then fitted to estimate the associations between TCBI and myocardial injury severity below and above the candidate cutoff. In the piecewise model, TCBI was modeled as two linear segments separated by the selected cutoff. TCBI was divided by 100 before model fitting; therefore, the adjusted odds ratios represent the odds of being in a higher myocardial injury severity category per 100-unit increase in TCBI within each segment. The two-piecewise model was compared with the conventional linear model using a likelihood ratio test. All models were adjusted for age, sex, hypertension, diabetes mellitus, hyperlipidemia, coronary artery disease, atrial fibrillation history, stroke history, smoking history, baseline NIHSS score, baseline ASPECTS, occlusion site, TOAST classification, final mTICI score, first-line thrombectomy technique, and BMI.

Ordered logistic regression models were used to investigate the association between TCBI and troponin trajectory (treated as an ordinal outcome: no injury < non-dynamic elevation < dynamic elevation). Given that BMI is a widely used surrogate for nutritional and metabolic status, its inclusion as a covariate was considered essential. Because TCBI incorporates body weight, which is closely related to BMI, adjustment for BMI may introduce collinearity or overadjustment. Therefore, we assessed the correlation between low TCBI and BMI using biserial correlation analysis before model construction, as low TCBI was defined as a dichotomous variable derived from a continuous measure. In the absence of significant multicollinearity, BMI was retained in all multivariable models. Three sequential adjustment models were constructed. Model 1 was adjusted for BMI alone to isolate its confounding effect. Model 2 further incorporated demographic characteristics (age, sex), vascular risk factors (hypertension, diabetes mellitus, hyperlipidemia, CAD, AF history, prior stroke history, and smoking history), and BMI. Given that stroke-related variables, including baseline stroke severity and imaging characteristics, are critical determinants of neurological injury and functional recovery, Model 3 extended Model 2 by additionally adjusting for baseline NIHSS score, ASPECTS, occlusion site, TOAST classification, first-line thrombectomy technique, and final mTICI grade. These three models were applied to evaluate the independent associations between TCBI and both troponin trajectory and 90-day poor functional outcome (mRS 3–6), with results expressed as odds ratios (ORs) with 95% confidence intervals (CIs). Multicollinearity was further evaluated across all three models using variance inflation factors (VIF) and tolerance values. ROC curve analysis compared discriminative performance of TCBI versus albumin, TG, and BMI using the DeLong test.

For subgroup analyses, the outcome was dichotomized into dynamic troponin elevation versus non-dynamic troponin elevation or no myocardial injury, given the distinct clinical implications of dynamic troponin elevation in this population. Subgroup analyses were performed across clinical characteristics, including sex, hypertension, diabetes mellitus, hyperlipidemia, CAD, AF, and stroke history. Within each stratum, the association between TCBI and the dichotomized outcome was evaluated using multivariable logistic regression.

Multivariable logistic regression was used to evaluate the association between myocardial injury pattern and 90-day poor functional outcome, adjusting for the same covariates as Model 3.

Mediation analysis was performed using the ‘mediation’ package in R. The total effect was decomposed into the average mediation effect (ACME, indirect effect) and the average direct effect (ADE). Point estimates and 95% CIs were derived using bootstrap resampling with 1,000 simulations.

To further validate the association and minimize residual confounding, propensity score matching (PSM) was performed using 1:1 nearest-neighbor matching without replacement, with a caliper width of 0.2 standard deviations of the logit of the propensity score. All statistical tests were two-sided, with *p* < 0.05 considered statistically significant. Analyses were performed using R software (version 4.3.2; R Foundation for Statistical Computing, Vienna, Austria).

## 3 Results

### 3.1 Baseline characteristics

From June 2021 through June 2024, a total of 668 consecutive patients with acute anterior circulation LVO who underwent EVT were initially screened from the stroke registries. Of these, 44 patients were excluded due to pre-stroke disability (mRS > 2) or age <18 years. To ensure the accuracy of nutritional and cardiac biomarkers, we further excluded 74 patients with confounding comorbidities, including active malignancy or severe infection (*n* = 35), severe renal insufficiency (*n* = 28), and ACS (*n* = 11). Additionally, 26 patients were excluded due to missing data for TCBI components, representing 3.9% of the initially screened cohort. Given the low proportion of missing data and the fact that complete lipid panels and serial cardiac biomarkers are essential for the core exposure and mediator variables in this study, complete-case analysis was deemed appropriate; multiple imputation was not applied as the missing fraction was unlikely to introduce substantial selection bias.

**Figure 1:**
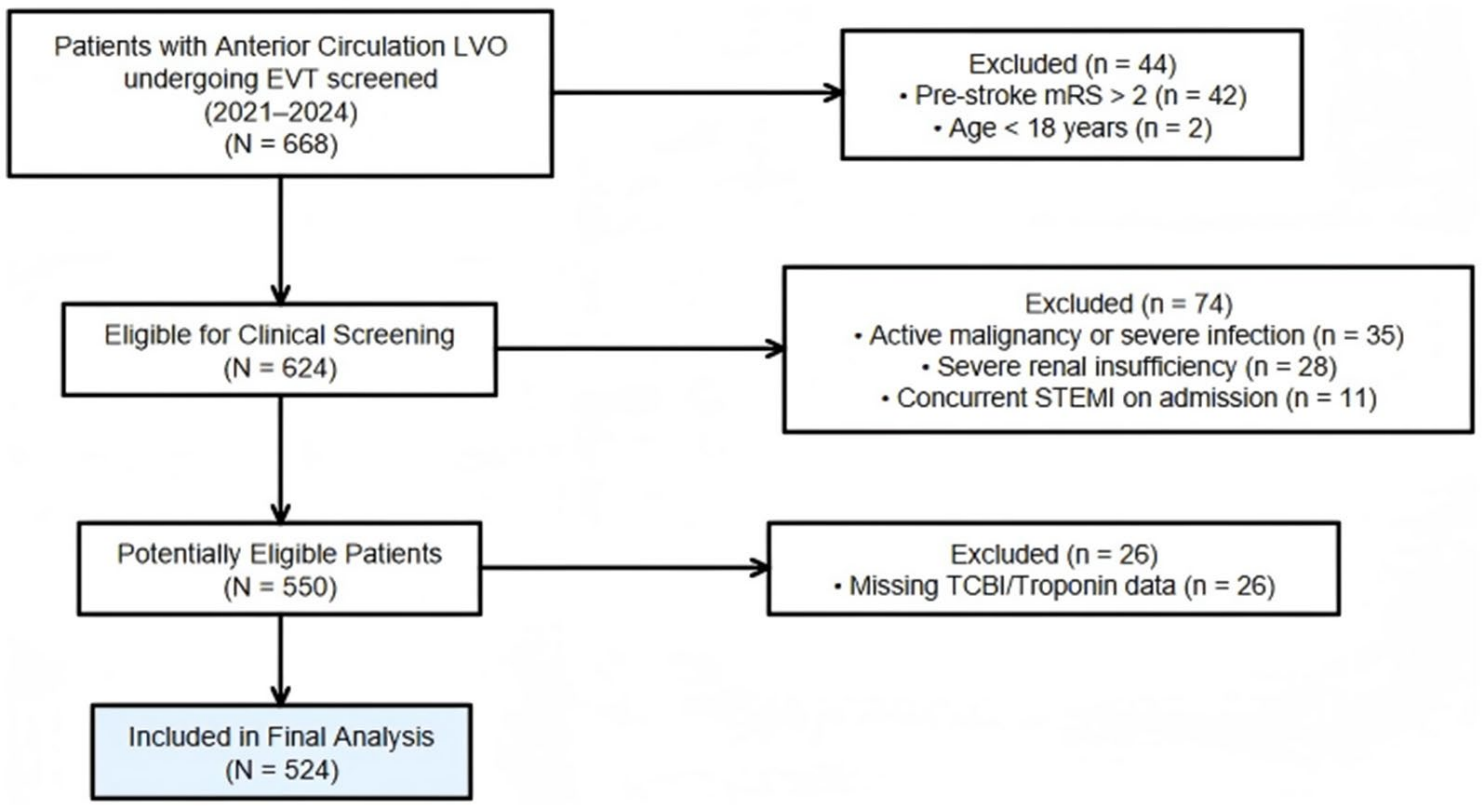
Patient selection flowchart.

Ultimately, 524 patients were included in the final analysis (Figure 1). The high inclusion rate (78.4%) ensures the representativeness of the study cohort. A total of 524 patients with acute anterior circulation LVO stroke who underwent EVT were included. Patients were categorized into three groups: no myocardial injury (*n* = 289, 55.2%), non-dynamic elevation (*n* = 76, 14.5%), and dynamic elevation (*n* = 159, 30.3%). Baseline characteristics are presented in Table 1.

**Table 1:** Baseline characteristics of study population stratified by myocardial injury pattern.

Characteristic	No myocardial injury (*N* = 289)	Non-dynamic elevation (*N* = 76)	Dynamic elevation (*N* = 159)	*p* value
Age, years, median (IQR)	67 (57, 74)	71 (62, 77)	73 (66, 80)	<0.001
Female sex, n (%)	107 (37.0)	36 (47.4)	77 (48.4)	0.038
Body mass index, kg/m^2^, median (IQR)	26.4 (23.3, 30.0)	27.5 (24.6, 30.7)	25.0 (22.5, 29.1)	0.036
Baseline NIHSS score, median (IQR)	14 (8, 21)	19 (12, 25)	21 (15, 30)	<0.001
Occlusion site, n (%)				0.817
Internal carotid artery	148 (51.2)	43 (56.6)	88 (55.3)	
M1 segment of MCA	107 (37.0)	24 (31.6)	51 (32.1)	
M2 segment of MCA	34 (11.8)	9 (11.8)	20 (12.6)	
Coronary artery disease, n (%)	47 (16.3)	24 (31.6)	58 (36.5)	<0.001
TOAST classification, n (%)				0.002
Cardioembolism	103 (35.6)	34 (44.7)	86 (54.1)	
Large-artery atherosclerosis	157 (54.3)	37 (48.7)	57 (35.8)	
Other or undetermined etiology	29 (10.0)	5 (6.6)	16 (10.1)	
Hypertension, n (%)	185 (64.0)	51 (67.1)	117 (73.6)	0.118
Diabetes mellitus, n (%)	72 (24.9)	22 (28.9)	53 (33.3)	0.162
Hyperlipidemia, n (%)	67 (23.2)	18 (23.7)	43 (27.0)	0.652
Atrial fibrillation history, n (%)	102 (35.3)	30 (39.5)	75 (47.2)	0.048
Stroke history, n (%)	53 (18.3)	19 (25.0)	25 (15.7)	0.229
Smoking history, n (%)	85 (29.4)	17 (22.4)	31 (19.5)	0.056
Baseline ASPECTS, median (IQR)	8.0 (6.0, 9.0)	7.0 (4.0, 9.0)	7.0 (4.0, 8.0)	<0.001
Final mTICI 2b-3, n (%)	272 (94.1)	67 (88.2)	148 (93.1)	0.196
First-line thrombectomy technique, n (%)				0.376
No thrombectomy	27 (9.3)	6 (7.9)	7 (4.4)	
Contact aspiration	75 (26.0)	25 (32.9)	48 (30.2)	
Stent retriever	17 (5.9)	5 (6.6)	6 (3.8)	
Combined technique	170 (58.8)	40 (52.6)	98 (61.6)	
Symptomatic intracranial hemorrhage, n (%)	18 (6.2)	9 (11.8)	19 (11.9)	0.073
Malignant cerebral edema, n (%)	55 (19.0)	17 (22.4)	51 (32.1)	0.008
Albumin, g/L, median (IQR)	39.0 (36.6, 42.0)	40.0 (35.8, 42.2)	38.0 (35.0, 41.4)	0.035
TG, mg/dL, median (IQR)	110 (81, 159)	118 (78, 180)	97 (66, 156)	0.029
TC, mg/dL, median (IQR)	163 (145, 200)	166 (145, 195)	173 (140, 203)	0.767
TCBI, median (IQR)	1,329 (929, 2083)	1,103 (758, 1734)	1,057 (685, 1930)	0.001

NIHSS, National Institutes of Health Stroke Scale; MCA, middle cerebral artery; ASPECTS, Alberta Stroke Program Early CT Score; TOAST, Trial of Org 10,172 in Acute Stroke Treatment; mTICI, modified Thrombolysis in Cerebral Infarction; TG, triglyceride; TC, total cholesterol; TCBI, triglyceride-cholesterol-body weight index [calculated as TG (mg/dL) × TC (mg/dL) × BW (kg) / 1,000].

Compared with patients without myocardial injury, those with dynamic elevation were older, had higher baseline NIHSS scores, and lower ASPECTS. CAD and AF were more prevalent in the dynamic elevation group. Lipid profiles and nutritional status also differed among groups, with lower TG, TCBI levels observed in the dynamic elevation group. Final recanalization rates (mTICI 2b-3) were high and comparable across groups. Malignant cerebral edema occurred more frequently in the dynamic elevation group.

### 3.2 Association between TCBI and myocardial injury

**Figure 2:**
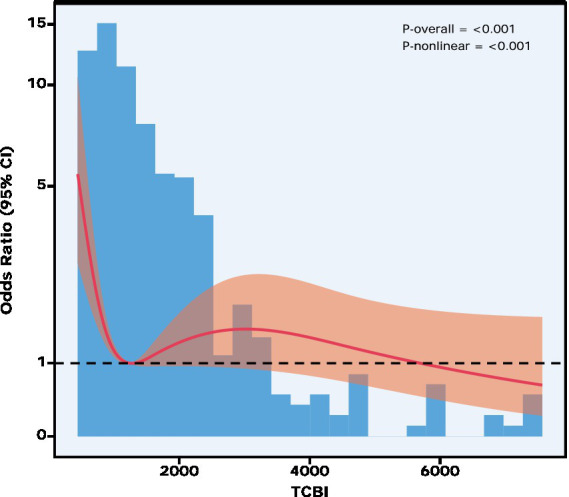
Non-linear association between TCBI and myocardiali injury risk.

RCS analysis showed a significant overall association between TCBI and myocardial injury severity (P overall < 0.001), with evidence of nonlinearity (P nonlinearity < 0.001; Figure 2). The RCS curve of nonlinearity (P nonlinearity < 0.001; Figure 2). The RCS curve showed a nonlinear dose-response pattern and crossed the null reference line at approximately TCBI = 1244. The 95% confidence interval widened at higher TCBI values, corresponding to the sparse distribution of observations in this range.

Based on this RCS-derived crossing point and previously reported threshold ranges, TCBI = 1244 was selected as the candidate cutoff for threshold analysis. In the two-piecewise ordered logistic regression model, TCBI was inversely associated with myocardial injury severity below the cutoff (TCBI < 1244: aOR = 0.88, 95% CI: 0.82–0.95, *p* = 0.001), whereas the association was not statistically significant above the cutoff (TCBI > 1244: aOR = 0.99, 95% CI: 0.98–1.03, *p* = 0.913; Supplementary Table S1). The two-piecewise model provided a better fit than the conventional linear model, as indicated by the likelihood ratio test (χ^2^(1) = 8.73, *p* = 0.003).

Biserial correlation analysis revealed no meaningful association between Low TCBI and BMI (*ρ* = 0.004, 95% CI: −0.082 to 0.089, *p* = 0.932; Supplementary Table S3). Ordered logistic regression analysis across all three models consistently showed that low TCBI (TCBI ≤ 1,244) was associated with higher odds of more severe myocardial injury (Table 2). In the fully adjusted model (Model 3), patients with low TCBI had 1.67 times the odds of more severe myocardial injury compared to those with high TCBI (OR = 1.67, 95% CI: 1.13–2.47, *p* = 0.010; Figure 2).

**Table 2:** Association between low TCBI and myocardial injury severity: ordered logistic regression analysis.

Analysis method	OR	95% CI	*p* value
Model 1	1.64	1.17–2.27	0.004
Model 2 (demographic and comorbidity adjusted)	1.51	1.05–2.17	0.027
Model 3 (fully adjusted)	1.67	1.13–2.47	0.010
OR, odds ratio; CI, confidence interval; TCBI, triglyceride-total cholesterol-bodyweight index.			

Model 1: Adjusted for BMI. Model 2: Adjusted for age, sex, hypertension, diabetes mellitus, hyperlipidemia, coronary artery disease, atrial fibrillation history, stroke history, smoking history, and BMI. Model 3: Additionally adjusted for baseline NIHSS score, baseline ASPECTS, occlusion site, TOAST classification, final mTICI, first-line thrombectomy technique, and BMI.

### 3.3 Subgroup analyses

In the overall cohort, Low TCBI was associated with dynamic troponin elevation (aOR = 1.59, 95% CI: 1.02–2.49, *p* = 0.040). Point estimates were in the same direction across subgroups defined by sex, hypertension, diabetes mellitus, hyperlipidemia, CAD, AF history, and stroke history. P-interaction values were non-significant for all subgroups (all P-interaction > 0.05; Supplementary Figure S1).

### 3.4 Correlation of TCBI with 90-day functional outcomes

Low TCBI (TCBI ≤ 1,244) was associated with 90-day poor functional outcome (mRS 3–6) in all three models (Table 3). The OR was 1.48 (95% CI: 1.04–2.10, *p* = 0.028) in Model 1, 1.47 (95% CI: 1.02–2.12, *p* = 0.039) in Model 2, and 1.54 (95% CI: 1.06–2.23, *p* = 0.024) in Model 3. VIF values were below 2.0 and tolerance values exceeded 0.50 across all three models (Supplementary Table S4).

**Table 3:** Association between TCBI and 90-day poor functional outcome.

Characteristic	Model 1			Model 2			Model 3		
	OR	95% CI	*p*-value	OR	95% CI	*p*-value	OR	95% CI	*p*-value
TCBI									
TCBI >1,244	—	—		—	—		—	—	
TCBI ≤1,244	1.48	1.04, 2.10	0.028	1.47	1.02, 2.12	0.039	1.54	1.06, 2.23	0.024

Model 1: Adjusted for BMI. Model 2: Adjusted for age, sex, hypertension, diabetes mellitus, hyperlipidemia, coronary artery disease, atrial fibrillation history, stroke history, and smoking history and BMI. Model 3: Additionally adjusted for baseline NIHSS score, baseline ASPECTS, occlusion site, TOAST classification, final mTICI, first-line thrombectomy technique and BMI.

**Figure 3:**
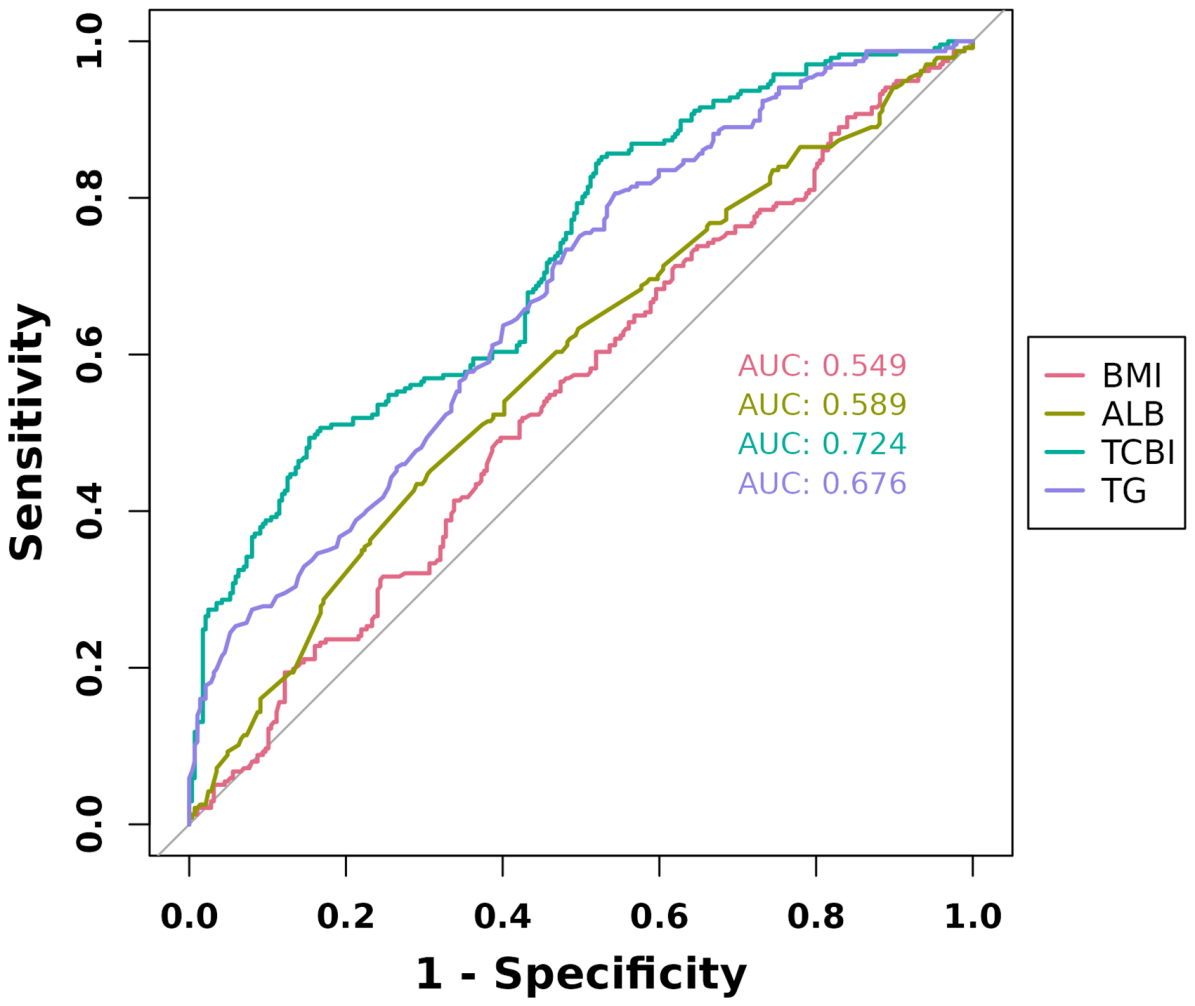
Receiver operating characteristic curves for predicting day poor functional outcome.

In ROC curve analysis, TCBI showed the highest discriminative ability for poor functional outcome, with an AUC of 0.723 (95% CI: 0.680–0.766). The AUCs for albumin, TG, and BMI were 0.589, 0.675, and 0.548, respectively, all of which were significantly lower than that of TCBI according to the DeLong test (all *p* < 0.001; Figure 3).

### 3.5 Association between TCBI and safety outcomes

The associations between high TCBI and safety outcomes are presented in Supplementary Tables S5, S6. In logistic regression analysis, no significant association was observed between high TCBI and malignant cerebral edema in any of the three models (Model 1: OR = 0.73, 95% CI: 0.49–1.10, *p* = 0.133; Model 2: OR = 0.83, 95% CI: 0.54–1.29, *p* = 0.411; Model 3: OR = 0.82, 95% CI: 0.53–1.27, *p* = 0.369). Similarly, no significant association was observed between high TCBI and sICH across all three models (Model 1: OR = 0.83, 95% CI: 0.45–1.53, *p* = 0.556; Model 2: OR = 0.90, 95% CI: 0.48–1.69, *p* = 0.740; Model 3: OR = 0.92, 95% CI: 0.49–1.74, *p* = 0.797).

### 3.6 Association between myocardial injury pattern and 90-day functional outcome

The association between troponin trajectory patterns and 90-day functional outcomes is presented in Table 4. After adjusting for confounders of Model 3, dynamic elevation of troponin was associated with poor 90-day functional outcomes (aOR 14.59, 95% CI: 8.35-25.49, *p*<0.001) compared to patients without myocardial injury. In contrast, non-dynamic elevation showed no significant association with poor outcomes (aOR 1.53, 95% CI: 0.87-2.70, *p*=0.142).

**Table 4:** Association between myocardial injury pattern and 90-day functional outcome.

Outcome	Group 1 (No myocardial injury)	Group 2 (Non-dynamic elevation)		Group 3 (Dynamic elevation)	
		aOR (95% CI)	*p*	aOR (95% CI)	*p*
90-day poor functional outcome	Ref	1.39(0.70, 2.77)	0.461	8.73(4.53, 16.83)	<0.001

### 3.7 Mediation analysis: myocardial injury as a potential mediator of the association between low TCBI and 90-day functional outcome

**Figure 4:**
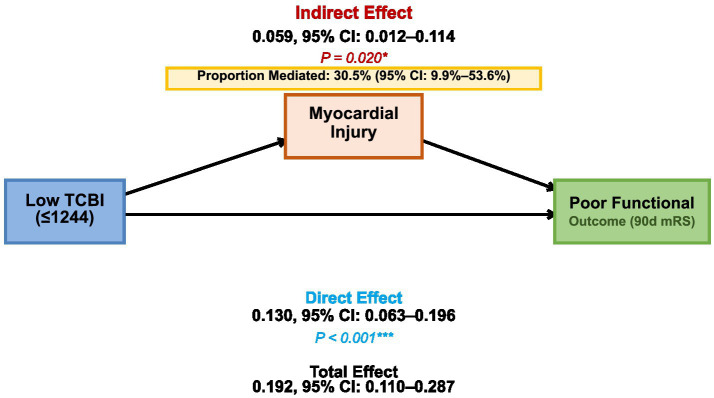
Mediation analysis.

A mediation analysis was performed using bootstrap resampling with 1,000 iterations (Figure 4). The total effect of low TCBI on poor functional outcome was 0.192 (95% CI: 0.110–0.287, *p* < 0.001). The indirect effect (ACME) through myocardial injury was 0.059 (95% CI: 0.012–0.114, *p* = 0.020), and the direct effect (ADE) was 0.134 (95% CI: 0.063–0.196, *p* < 0.001). The estimated proportion mediated was 30.5% (95% CI: 9.9%–53.6%).

### 3.8 Sensitivity analysis

PSM was performed using 1:1 nearest-neighbor matching without replacement (caliper width = 0.2 standard deviations of the logit of the propensity score), yielding 191 matched pairs (matching rate: 382/524, 72.9%). Baseline characteristics before and after matching are presented in Supplementary Table S7. After matching, all standardized mean differences were below 0.1.

In the matched cohort, Low TCBI was associated with more severe myocardial injury (OR = 1.50, 95% CI: 1.01–2.22, *p* = 0.042) and 90-day poor functional outcome (OR = 1.52, 95% CI: 1.04–2.23, *p* = 0.032; Supplementary Table S8). In the matched cohort, dynamic troponin elevation was associated with 90-day poor functional outcome (crude OR = 6.67, 95% CI: 3.59–12.38, *p* < 0.001), whereas non-dynamic elevation was not (crude OR = 1.47, 95% CI: 0.44–4.92, *p* = 0.534; Supplementary Table S9).

## 4 Discussion

In this two-center cohort study of patients with LVO-AIS who received EVT, we found that Low TCBI was independently associated with both dynamic myocardial injury and 90-day poor functional outcome. Mediation analysis suggested that myocardial injury may partly account for the association between Low TCBI and poor functional outcome, with an estimated proportion mediated of 45.6%. To our knowledge, few studies have examined the interplay between nutritional-metabolic status, stroke-associated myocardial injury, and functional outcome specifically in the context of mechanical thrombectomy.

Evaluating the nutritional status of patients in the acute phase of severe stroke is highly challenging. Traditional screening tools such as NRS-2002 or MNA rely on medical history collection, but in patients with LVO, this approach is often not feasible due to aphasia or consciousness disorders (11, 26). Although the Geriatric Nutritional Risk Index (GNRI) is a valuable objective tool (27, 28), it heavily depends on serum albumin. As previously pointed out in a study on cardiac intensive care patients (11), albumin is a negative acute-phase reactant; during EVT, its level may rapidly decline due to systemic inflammation or fluid resuscitation, which may lead to misjudgment of nutritional status. In contrast, TCBI incorporates TG, TC, and body weight - parameters that are relatively stable and easily accessible in the hyperacute phase. Our research findings are consistent with multiple previous reports, confirming that TCBI can serve as a reliable “anti-stress” metabolic indicator for hemodynamically unstable patients (11, 29).

We observed that lower TCBI was associated with poorer prognosis, supporting the “lipid paradox” commonly seen in critically ill patients. In acute conditions such as stroke, metabolic indicators often show reversed prognostic implications compared to chronic disease states (6, 8). Although hyperlipidemia is a risk factor for atherosclerosis, extremely low cholesterol levels are associated with larger infarct volumes and higher mortality in acute stroke (7, 30). The threshold of TCBI = 1,244 identified in the present study is consistent with values reported in related populations. A restricted cubic spline analysis in a Korean AIS cohort of 1,764 patients identified an inflection point of 1227.3 (31), and an optimal cutoff of 1112.37 was reported for predicting stroke-associated pneumonia in a Chinese AIS cohort (32). These converging thresholds across independent cohorts with different ethnicities and clinical contexts suggest a degree of cross-population stability for this range. Nevertheless, as the threshold was derived empirically from the present cohort using a data-driven grid-search approach, caution is warranted when applying this specific value to other populations or institutions where differences in laboratory assay methods, unit conventions (e.g., mg/dL vs. mmol/L), or patient demographics may systematically shift absolute TCBI values. External validation in prospective, multicenter cohorts is needed before this threshold can be adopted as a generalizable clinical decision boundary.

One notable finding of our study is the potential mediating role of myocardial injury in the association between low TCBI and unfavorable functional outcomes. Zhang et al. previously linked GNRI to myocardial injury (27), yet the downstream pathway to functional outcomes had remained unclear. Our mediation analysis suggests that in patients with depleted metabolic reserves, acute stress-related myocardial injury may represent one plausible pathway through which low nutritional status contributes to poor outcomes, possibly via hemodynamic instability and impaired cerebral perfusion following recanalization. However, given the retrospective and observational nature of this study, this mediation analysis should be interpreted as exploratory rather than confirmatory.

These findings offer several considerations for clinical management. Although statins remain the cornerstone of secondary prevention in AIS (33, 34), the optimal lipid-lowering strategy in patients with low nutritional reserves warrants careful individualization, particularly in elderly or frail patients (8). For patients with LVO-AIS and low TCBI, clinicians may consider: (1) prioritizing early nutritional support to replenish metabolic reserves (5); (2) implementing enhanced cardiac monitoring to facilitate timely detection of stroke-associated myocardial injury (27). Regarding lipid-lowering therapy, given that pre-stroke statin use was unavailable in the present study and may have influenced TCBI values through artificial reduction of TC levels, no definitive recommendations regarding statin management can be drawn from the current data. Future studies incorporating complete medication records will be needed to clarify the interaction between lipid-lowering therapy, nutritional status, and stroke outcomes.

This study is a retrospective analysis and has inherent limitations. Firstly, the exclusion of patients with active malignancy, severe infection, severe renal insufficiency, and concurrent ACS, while methodologically necessary to ensure the specificity of TCBI and troponin measurements, may limit the generalizability of our findings to these subpopulations, which are not uncommon in real-world stroke practice. Secondly, we lack comprehensive baseline cardiac assessment data, including left ventricular ejection fraction (LVEF), detailed cardiac structural disease evaluation, and levels of natriuretic peptides (BNP/NT-proBNP). Although we adjusted for the history of CAD and AF, the lack of quantitative cardiac function parameters limits our ability to fully distinguish between pre-existing cardiac dysfunction and injury caused by acute stroke. Future studies that incorporate systematic cardiac ultrasound examinations and cardiac biomarkers will be able to describe the baseline cardiac status in greater detail. Thirdly, we acknowledge limitations related to biomarker timing. Fasting samples were collected at a median of 17 h post-admission, after EVT completion. Although TG, TC, and body weight are relatively resistant to acute inflammatory changes, albumin may have already declined due to post-procedural inflammation, which may have partly attenuated its predictive performance and should be considered when interpreting the AUC comparison. More broadly, TCBI measured in the acute phase may reflect both chronic metabolic status and acute stress responses; although we adjusted for multiple severity markers and applied strict fasting protocols, acute stress contributions cannot be completely excluded—a fundamental interpretative challenge for any biomarker measured in the acute phase (35). Furthermore, data on pre-stroke lipid-lowering therapy, particularly statin use, were unavailable in the present study. As statins may artificially reduce TC levels, TCBI values in treated patients may have been underestimated, potentially introducing misclassification bias. Future studies with complete medication records would allow adjustment for this confounding factor.

## 5 Conclusion

In LVO-AIS patients undergoing EVT, low TCBI (≤1,244) was independently associated with more severe stroke-associated myocardial injury and poor 90-day functional outcomes, with a nonlinear threshold effect identified. Exploratory mediation analysis suggested that myocardial injury partially mediates the relationship between depleted metabolic-nutritional reserves and unfavorable functional recovery (45.6%). Prospective studies with nutritional intervention protocols and systematic cardiac monitoring are warranted to validate these findings and establish causal relationships.

## Data Availability

The raw data supporting the conclusions of this article will be made available by the authors, without undue reservation.
