# Supplementary material for: A novel nutritional index for predicting stroke-heart syndrome and clinical outcomes after endovascular treatment
Source: Front Nutr. 2026 May 18;13:1822338. doi: 10.3389/fnut.2026.1822338 (PMC13231399; doi:10.3389/fnut.2026.1822338)
Supplement: Supplementary file 1 [file Table_1.docx]

Supplementary Material

**Figure S1:**Subgroup Analysis of the Association Between Low TCBI and Myocardial Injury


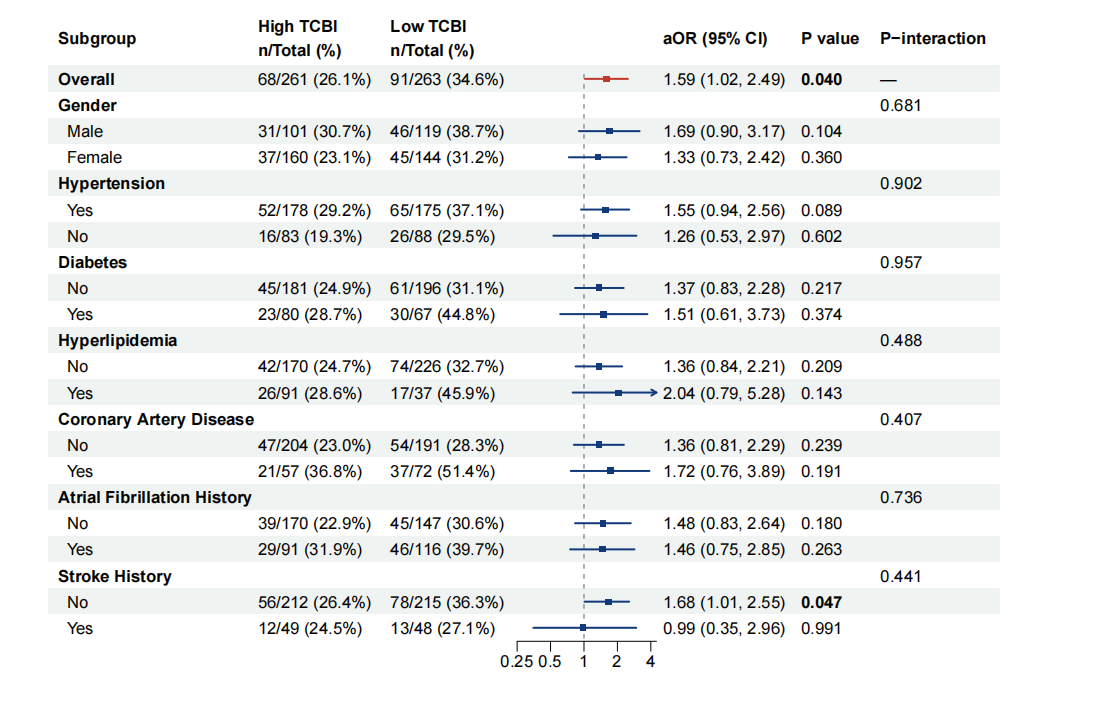


Table S1. Piecewise Ordered Logistic Regression: Threshold Effect Analysis of TCBI on Myocardial Injury Severity

| TCBI Range | aOR | 95% CI | P Value |
| --- | --- | --- | --- |
| ≤ 1244 (per 100-unit increase) | 0.88 | 0.82 – 0.95 | < 0.001 |
| > 1244 (per 100-unit increase) | 0.99 | 0.98 – 1.03 | 0.913 |
| Abbreviations: aOR, adjusted odds ratio; CI, confidence interval; TCBI, triglyceride-total cholesterol-bodyweight index. Threshold specification: TCBI = 1244 was selected as the candidate cutoff based on the approximate crossing point of the restricted cubic spline-estimated odds ratio curve with the null reference line and its consistency with previously reported threshold ranges. Model comparison: The two-piecewise ordered logistic regression model using TCBI = 1244 as the candidate cutoff provided a better fit than the conventional linear model, as assessed by the likelihood ratio test, χ²(1) = 8.73, P = 0.003. Unit: The aOR represents the odds ratio for being in a higher myocardial injury severity category per 100-unit increase in TCBI within each segment. TCBI was rescaled by dividing by 100 before model fitting. Adjustment: Models were adjusted for age, sex, hypertension, diabetes mellitus, hyperlipidemia, coronary artery disease, atrial fibrillation history, stroke history, smoking history, baseline NIHSS score, baseline ASPECTS, occlusion site, TOAST classification, final mTICI score, first-line thrombectomy technique, and BMI. | | | |

Table S2. Biserial Correlation Between TCBI and BMI

| Variable 1 | Variable 2 | ρ (rho) | 95% CI | CI Low | CI High | t | df | p-value |
| --- | --- | --- | --- | --- | --- | --- | --- | --- |
| TCBI | BMI | 0.004 | [-0.082, 0.089] | -0.082 | 0.089 | 0.086 | 522 | 0.932 |
| Method: Biserial correlation \| n = 524 \| Confidence level: 95% | | | | | | | | |
| Note: Biserial correlation is the appropriate method when one variable is dichotomous and derived from an | | | | | | | | |
| underlying continuous distribution (i.e., Low TCBI was defined as TCBI ≤ 1244). | | | | | | | | |
| ρ ≈ 0 and p = 0.932 indicate no meaningful association between Low TCBI and BMI, | | | | | | | | |
| confirming the absence of multicollinearity between these two variables in the multivariable models. | | | | | | | | |

Table S3. Multicollinearity Diagnostics for Multivariable Regression Models (Models 1–3)

| Term | Model 1 | | Model 2 | | Model 3 | |
| --- | --- | --- | --- | --- | --- | --- |
|  | VIF | Tolerance | VIF | Tolerance | VIF | Tolerance |
| TCBI | 1.000 | 1.000 | 1.089 | 0.918 | 1.110 | 0.901 |
| Sex | — | — | 1.422 | 0.703 | 1.466 | 0.682 |
| Age | — | — | 1.198 | 0.835 | 1.233 | 0.811 |
| Occlusion site | — | — | — | — | 1.105 | 0.905 |
| TOAST classification | — | — | — | — | 1.565 | 0.639 |
| Hypertension | — | — | 1.032 | 0.969 | 1.064 | 0.940 |
| Diabetes mellitus | — | — | 1.057 | 0.946 | 1.061 | 0.943 |
| Hyperlipidemia | — | — | 1.171 | 0.854 | 1.228 | 0.815 |
| Coronary artery disease | — | — | 1.105 | 0.905 | 1.120 | 0.893 |
| Atrial fibrillation history | — | — | 1.142 | 0.875 | 1.693 | 0.591 |
| Smoking history | — | — | 1.399 | 0.715 | 1.457 | 0.686 |
| Stroke history | — | — | 1.021 | 0.979 | 1.052 | 0.950 |
| Baseline NIHSS score | — | — | — | — | 1.234 | 0.810 |
| Final mTICI | — | — | — | — | 1.158 | 0.863 |
| First-line thrombectomy technique | — | — | — | — | 1.129 | 0.886 |
| Baseline ASPECTS | — | — | — | — | 1.193 | 0.838 |
| BMI | 1.000 | 1.000 | 1.028 | 0.973 | 1.039 | 0.962 |

Multicollinearity was assessed using variance inflation factors (VIF) and tolerance values for all covariates included in Models 1 through 3. VIF < 5 and tolerance > 0.20 were considered acceptable thresholds; values below 2.0 and above 0.50, respectively, indicate no meaningful multicollinearity. Abbreviations: VIF, variance inflation factor; TCBI, triglyceride-total cholesterol-bodyweight index; BMI, body mass index; NIHSS, National Institutes of Health Stroke Scale; ASPECTS, Alberta Stroke Program Early CT Score; TOAST, Trial of Org 10172 in Acute Stroke Treatment; mTICI, modified Thrombolysis in Cerebral Infarction.

Table S4. Association Between Low TCBI and Malignant Cerebral Edema: Logistic Regression Analysis

| Characteristic | Model 1 | | | Model 2 | | | Model 3 | | |
| --- | --- | --- | --- | --- | --- | --- | --- | --- | --- |
|  | OR | 95% CI | p-value | OR | 95% CI | p-value | OR | 95% CI | p-value |
| TCBI |  |  |  |  |  |  |  |  |  |
| low | — | — |  | — | — |  | — | — |  |
| High | 0.73 | 0.49, 1.10 | 0.133 | 0.83 | 0.54, 1.29 | 0.411 | 0.82 | 0.53, 1.27 | 0.369 |
| Abbreviations: CI = Confidence Interval, OR = Odds Ratio | | | | | | | | | |
| Model 1: Adjusted for BMI. | | | | | | | | | |
| Model 2: Adjusted for age, sex, hypertension, diabetes mellitus, hyperlipidemia, coronary artery disease, atrial fibrillation history, stroke history, smoking history, and BMI. | | | | | | | | | |
| Model 3: Additionally adjusted for baseline NIHSS score, baseline ASPECTS, occlusion site, TOAST classification, final mTICI, first-line thrombectomy technique, and BMI. | | | | | | | | | |

Table S5. Association Between Low TCBI and Symptomatic Intracranial Hemorrhage: Logistic Regression Analysis

| Characteristic | Model 1 | | | Model 2 | | | Model 3 | | |
| --- | --- | --- | --- | --- | --- | --- | --- | --- | --- |
|  | OR | 95% CI | p-value | OR | 95% CI | p-value | OR | 95% CI | p-value |
| TCBI |  |  |  |  |  |  |  |  |  |
| low | — | — |  | — | — |  | — | — |  |
| High | 0.83 | 0.45, 1.53 | 0.556 | 0.90 | 0.48, 1.69 | 0.740 | 0.92 | 0.49, 1.74 | 0.797 |
| Abbreviations: CI = Confidence Interval, OR = Odds Ratio | | | | | | | | | |
| Model 1: Adjusted for BMI. | | | | | | | | | |
| Model 2: Adjusted for age, sex, hypertension, diabetes mellitus, hyperlipidemia, coronary artery disease, atrial fibrillation history, stroke history, smoking history, and BMI. | | | | | | | | | |
| Model 3: Additionally adjusted for baseline NIHSS score, baseline ASPECTS, occlusion site, TOAST classification, final mTICI, first-line thrombectomy technique, and BMI. | | | | | | | | | |

Table S6. Baseline Characteristics Before and After Propensity Score Matching

| **Variable** | **Before Matching** | | | **After Matching** | | |
| --- | --- | --- | --- | --- | --- | --- |
|  | **TCBI ≤1244**  **(n = 263)** | **TCBI >1244**  **(n = 261)** | **SMD** | **TCBI ≤1244**  **(n = 191)** | **TCBI >1244**  **(n = 191)** | **SMD** |
| **Demographics** | | | | | | |
| Age, years | 69 ± 12 | 66 ± 12 | **0.245** | 68 ± 13 | 67 ± 12 | **0.011** |
| Female sex | 119 (45.2) | 101 (38.7) | **0.132** | 76 (39.8) | 77 (40.3) | **0.011** |
| BMI, kg/m² | 26.9 ± 4.8 | 26.9 ± 5.3 | **0.006** | 26.8 ± 4.7 | 27.0 ± 5.4 | **0.042** |
| **Stroke characteristics** | | | | | | |
| Baseline NIHSS score | 19 ± 9 | 17 ± 10 | **0.114** | 19 ± 9 | 18 ± 10 | **0.057** |
| Occlusion site |  |  |  |  |  |  |
| Internal carotid artery | 136 (51.7) | 143 (54.8) | **0.062** | 104 (54.5) | 103 (53.9) | **0.010** |
| M1 segment of MCA | 96 (36.5) | 86 (33.0) | **0.074** | 66 (34.6) | 65 (34.0) | **0.011** |
| M2 segment of MCA | 31 (11.8) | 32 (12.3) | **0.015** | 21 (11.0) | 23 (12.0) | **0.032** |
| Baseline ASPECTS | 8.00 (5.00, 9.00) | 8.00 (6.00, 9.00) | **0.021** | 8.00 (7.00, 9.00) | 8.00 (7.00, 9.00) | **0.000** |
| TOAST classification |  |  |  |  |  |  |
| Cardioembolism | 129 (49.0) | 94 (36.0) | **0.261** | 76 (39.8) | 77 (40.3) | **0.010** |
| Large-artery atherosclerosis | 109 (41.4) | 142 (54.4) | **0.263** | 97 (50.8) | 94 (49.2) | **0.032** |
| Other/undetermined etiology | 25 (9.5) | 25 (9.6) | **0.002** | 18 (9.4) | 20 (10.5) | **0.036** |
| **Comorbidities** | | | | | | |
| Hypertension | 175 (66.5) | 178 (68.2) | **0.035** | 129 (67.5) | 124 (64.9) | **0.055** |
| Diabetes mellitus | 67 (25.5) | 80 (30.7) | **0.119** | 47 (24.6) | 49 (25.7) | **0.024** |
| Hyperlipidemia | 37 (14.1) | 91 (34.9) | **0.598** | 37 (19.4) | 29 (15.2) | **0.095** |
| Coronary artery disease | 72 (27.4) | 57 (21.8) | **0.124** | 41 (21.5) | 44 (23.0) | **0.035** |
| Atrial fibrillation history | 116 (44.1) | 91 (34.9) | **0.186** | 73 (38.2) | 75 (39.3) | **0.021** |
| Stroke history | 48 (18.3) | 49 (18.8) | **0.014** | 36 (18.8) | 34 (17.8) | **0.027** |
| Smoking history | 61 (23.2) | 72 (27.6) | **0.104** | 47 (24.6) | 48 (25.1) | **0.012** |
| **Treatment characteristics** | | | | | | |
| First-line thrombectomy technique |  |  |  |  |  |  |
| No thrombectomy | 16 (6.1) | 24 (9.2) | **0.130** | 15 (7.9) | 11 (5.8) | **0.088** |
| Contact aspiration | 83 (31.6) | 65 (24.9) | **0.143** | 57 (29.8) | 55 (28.8) | **0.023** |
| Stent retriever | 19 (7.2) | 9 (3.4) | **0.146** | 5 (2.6) | 9 (4.7) | **0.081** |
| Combined technique | 145 (55.1) | 163 (62.5) | **0.147** | 114 (59.7) | 116 (60.7) | **0.021** |
| Final mTICI 2b–3 | 244 (92.8) | 243 (93.1) | **0.013** | 177 (92.7) | 179 (93.7) | **0.040** |
| **Post-procedural complications** | | | | | | |
| Symptomatic intracranial hemorrhage | 25 (9.5) | 21 (8.0) | **0.050** | 16 (8.4) | 16 (8.4) | **0.000** |
| Malignant cerebral edema | 69 (26.2) | 54 (20.7) | **0.126** | 49 (25.7) | 45 (23.6) | **0.048** |
| Data are presented as mean ± SD or *n* (%). **Abbreviations:** SMD, standardized mean difference; TCBI, triglyceride-total cholesterol-bodyweight index; NIHSS, National Institutes of Health Stroke Scale; BMI, body mass index; MCA, middle cerebral artery; TOAST, Trial of Org 10172 in Acute Stroke Treatment; mTICI, modified Thrombolysis in Cerebral Infarction. Propensity score matching was performed using 1:1 nearest neighbor matching without replacement with a caliper width of 0.2 standard deviations of the logit of the propensity score. Matching variables included all covariates listed in the table. After matching, all covariates achieved excellent balance (all SMD <0.1) Matching rate: 382/524 (72.9%). A total of 191 matched pairs were successfully created. | | | | | | |

**Table S7.** Sensitivity Analysis: Association Between Low TCBI and Outcomes in the Propensity Score-Matched Cohort

| **Analysis Method** | **Crude OR** | **95% CI** | **P Value** |
| --- | --- | --- | --- |
| Myocardial Injury Severity | 1.50 | 1.01, 2.22 | 0.042 |
| 90-Day Poor Functional Outcome | 1.52 | 1.04, 2.23 | 0.032 |

**Table S8**. Sensitivity Analysis: Association between Myocardial Injury Pattern and 90-day Functional Outcomes in Propensity Score-Matched Cohort

| Outcome | Group 1 (No myocardial injury) | Group 2  (Non-dynamic elevation) | | Group 3  (Dynamic elevation) | |
| --- | --- | --- | --- | --- | --- |
|  |  | Crude OR (95% CI) | P | Crude OR  (95% CI) | P |
| 90-day Poor Functional Outcome | Ref | 1.47 (0.44, 4.92) | 0.534 | 6.67 (3.59, 12.38) | <0.001 |

Crude odds ratios are reported, as propensity score matching had already achieved excellent covariate balance between groups (all standardized mean differences <0.1), rendering further multivariable adjustment unnecessary. Reference category: No myocardial injury (Group 1). Abbreviations: OR, odds ratio; CI, confidence interval.
